# Increased incidence of human West Nile and Usutu infections in Austria, 2024: analysis of data from 2009 to 2024

**DOI:** 10.2807/1560-7917.ES.2026.31.1.2500260

**Published:** 2026-01-08

**Authors:** David M Florian, Jeremy V Camp, Christof Jungbauer, Dirk Werber, Andreas Reich, Karin Stiasny, Stephan W Aberle, Judith H Aberle

**Affiliations:** 1 Center for Virology, Medical University of Vienna, Vienna, Austria; 2 Austrian Red Cross, Blood Service for Vienna, Lower Austria and Burgenland, Vienna, Austria; 3 Department for Transfusion Medicine, University Hospital, Paracelsus Medical University, Salzburg, Austria; 4 Austrian Agency for Health and Food Safety, Vienna, Austria

**Keywords:** West Nile virus, Usutu virus, Outbreak, Austria

## Abstract

**BACKGROUND:**

West Nile virus (WNV) and Usutu virus (USUV) outbreaks in Europe pose growing public health concerns. In Austria, human WNV and USUV infections occur nearly every year since 2009 with notable case number variations.

**AIM:**

We analysed annual incidences and spatiotemporal distributions of human WNV and USUV infections in Austria in 2009–2024.

**METHODS:**

Annual incidence rates of laboratory-confirmed WNV and USUV cases recorded through the national surveillance were calculated, stratified by age, sex, clinical presentation, exposure place and virus sequence.

**RESULTS:**

During 2009–2024, recorded case numbers were highest in 2024, with 37 WNV (19 male/18 female; median age: 62 years, range: 18–88) and 27 USUV infections (18 male/9 female; median age: 59 years, range: 20–69). Nineteen WNV cases developed West Nile neuroinvasive disease, while no USUV cases had neurological disease. Thirty-four of the WNV cases and all USUV cases were locally acquired. In northern Burgenland, an eastern Austrian region with an avian hotspot and only sporadic cases previously reported, WNV and USUV incidences respectively rose from averages of 0.6 and 1.0 per 100,000 in previous years to 6.6 and 4.2 per 100,000 in 2024. All 25 sequences analysed in 2024 from locally acquired WNV cases were of lineage 2. Among 15 USUV sequences, 14 belonged to the Europe-2 and one to the Africa-3 clade.

**CONCLUSION:**

Human WNV and USUV infection increases in a previously low-incidence region underscore their increasing public health impact in Austria. Strengthening surveillance and response measures is essential for early detection, guiding prevention efforts, and ensuring blood donor safety.

**Key public health message**

**What did you want to address in this study and why?**

West Nile virus (WNV) and Usutu virus (USUV) are transmitted to humans via mosquitoes or, when no precautions are in place, blood transfusion. While human infections with either virus can cause neurological disease, this is rare for USUV. Since the early 2000s, WNV has expanded to several new areas in Europe. We assessed annual incidences and geographical spread of human WNV cases in Austria from 2009 to 2024, and simultaneously those of USUV cases.

**What have we learnt from this study?**

During the study, the highest numbers of annual cases with each virus recorded were in 2024, with 37 WNV (34 autochthonous) and 27 USUV (all locally acquired) infections. Among the 37 WNV cases, 19 developed West Nile neuroinvasive disease. Infections with WNV and USUV notably increased in northern Burgenland, an eastern Austrian region that had previously reported only sporadic cases. In this region, 16 WNV and seven USUV cases occurred in 2024.

**What are the implications of your findings for public health?**

An increase in numbers of WNV infections in a prior less affected Austrian region combined with the high proportion of cases of severe disease in the country underscores the growing health impact of WNV. While continuous surveillance remains essential, further strengthening and enhancing clinical awareness in areas previously considered at low risk is crucial for early case detection, informing targeted prevention strategies and maintaining blood donor safety.

## Introduction

West Nile virus (WNV) and Usutu virus (USUV) are mosquito-borne flaviviruses that have become endemic in several European countries [1,2]. Infection by WNV can cause neuroinvasive disease, substantially affecting animal and human health, while infection by USUV is usually asymptomatic and only rarely leads to neuroinvasive disease in humans [2-6]. Both viruses are primarily transmitted by *Culex* mosquitoes, with multiple bird species serving as amplifying hosts [7]. Humans and other mammals, like equids, are occasionally infected and considered dead-end hosts that do not contribute to onward transmission [8]. However, WNV can be transmitted through blood transfusion and organ transplantation [9], prompting the implementation of mandatory blood donor screening in endemic regions [9,10]. Over the past decades, WNV lineage 2 has demonstrated a marked and sustained spread across southern and central Europe, with recent epidemiological data suggesting a northward expansion, evidenced by its emergence in Germany and the Netherlands since the end of the 2010s [6,11,12]. Climate change, particularly milder winters and warmer springs, is considered an important factor for the emergence of WNV and USUV, as these conditions enhance mosquito activity and virus transmission [11,13,14].

The rising risk of outbreaks in previously unaffected regions underlines the need to strengthen virus surveillance throughout Europe [15]. In Austria, human cases of WNV were first detected in 2009 [16]. Since then, WNV has continued to cause infections in humans and has become endemic in the eastern part of the country. Unlike WNV, which is subject to mandatory reporting, USUV is not notifiable in Austria or at the European level. Nevertheless, it has been detected in birds and mosquitoes since 2001 [17] and has been monitored in Austria since 2014 through the WNV surveillance programme in blood donors. This integrated surveillance has shown an increase in human USUV infections in 2018, with 18 cases detected among blood donors [18].

Surveillance data spanning 2009 to 2018 revealed considerable year-to-year variation in WNV activity, with the highest recorded number of human cases during this period occurring in 2018 [18]. The aim of the current study was to describe the annual incidences and spatiotemporal distribution of human WNV and USUV cases in Austria recorded by the national reference centre (NRC) of human arbovirus infections in Vienna and the national public health authorities during the period 2009–2024.

## Methods

### Notification and detection of West Nile and Usutu virus infections

We used laboratory-confirmed WNV cases reported to the nationwide epidemiological notification system. Diagnostic testing of symptomatic patients, who were suspected of WNV infection (i.e. with symptoms characteristic of West Nile fever or neuroinvasive disease (WNF/WNND)) was performed at the Center for Virology, Medical University of Vienna, which serves as the national reference laboratory for human arbovirus infections.

In addition, since 2014, blood donors in affected areas (the federal provinces of Vienna, Lower Austria, Burgenland) have been screened each year for WNV using nucleic acid testing (NAT; Cobas WNV assay; Roche, Rotkreuz, Switzerland) between June and November. Samples testing positive in the WNV-NAT, which also detects USUV, are reported to the nationwide epidemiological notification system and confirmed at the national reference laboratory.

The case definition for WNV infections used in Austria is the European Union (EU) case definition as outlined in the Commission Implementing Decision (EU) 2018/945 [19]. According to these criteria, a confirmed WNV case requires one of the following: (i) detection of WNV-specific nucleic acid in blood, cerebrospinal fluid (CSF), or urine; (ii) detection of WNV-specific IgM in CSF; or (iii) high-titre WNV-specific IgM in serum combined with detection of WNV-specific IgG, confirmed by virus-specific neutralisation testing.

To minimise potential issues related to cross-reactivity between USUV and WNV, we conducted USUV testing for all reported WNV cases.

Diagnosis of USUV infections in blood donors was based on a positive NAT result, confirmed by USUV-specific PCR or by USUV neutralisation assays [20].

Each confirmed case was contacted and data on clinical presentation, location of most likely exposure, and travel history were collected. WNV cases were clinically classified as WNND if signs of central nervous system (CNS) involvement occurred, or WNF in cases of symptomatic infection (acute fever, arthralgia/myalgia, fatigue, headache, rash) without clinical CNS involvement. Cases of USUV presenting with any symptoms (arthralgia/myalgia, fever, headache, rash) were classified as symptomatic, while confirmed WNV and USUV infections detected in individuals without clinical symptoms were classified as asymptomatic.

Confirmed cases were characterised as locally acquired (i.e. autochthonous cases), if they had no travel history outside Austria in the 14 days prior to symptom onset or blood donation; alternatively, they were qualified as imported cases.

### Nucleic acid amplification and sequencing

Routine PCR testing as part of diagnosis was performed as described previously [16]. In short, RNA extraction was performed using NucliSENS easyMAG extractor (bioMérieux, Marcy l’Etoile, France), followed by real-time TaqMan RT-PCR for WNV with primers located in the 3´-untranslated region (UTR) and RT-PCR for USUV as described in [21].

For WNV, amplification of a 294 nt portion of the C/prM gene fragments (beginning at position 270 relative to the reference strain AF404757) by RT-PCR and Sanger-sequencing was performed for lineage differentiation in local and imported cases throughout the study period. Sequences for phylogenetic analysis were obtained with three different nested conventional RT-PCRs for WNV to amplify an 1,880 nt region (beginning at position 9,112 in the reference strain NC_001563) using three overlapping primer sets [22-24], spanning 1,274 nt of the NS5 gene and some of the 3’ UTR. The amplicons were pooled and sequenced on an Illumina MiSeq, pre-processed with fastp (v0.23.4), and mapped to the reference strain Austria/2008_gh (KF179640) with minimap2 (v2.26). Primer sequences were removed from the ends of mapped reads with iVar trim and consensus was assembled with minimum depth of 100 and frequency threshold of 0.5 (iVar v1.4.2). 

For USUV clade determination and phylogenetic analysis, a conventional RT-PCR was used to amplify a 436 nt region (beginning at position 9,201 relative to the reference strain NC_006551) of the NS5 gene, and amplicons were sequenced by the Sanger method.

### Phylogenetic analysis

Maximum likelihood inference was used to construct phylogenetic trees of both WNV and USUV with a selection of reference sequences including deduplicated patient-derived sequences generated in this study. The reference sequences were whole genome sequences selected from GenBank, down-sampling to include no more than two sequences per country, per year, and deduplicating with BBMap’s ‘dedupe’ function. In addition, we included Austrian whole genome sequences previously derived from human-, bird- and mosquito-derived samples. Sequences were aligned with the fast progressive method in MAFFT (v7.515), and maximum likelihood consensus trees were generated with IQTree2 (v2.2.0.3) with 1,000 ultrafast bootstrap replicates using the Tamura−Nei (TN) + empirical base frequency (F) +  proportion of invariable sites (I) + rate heterogeneity with three categories (R3) and the TN equal frequencies (TNe) + gamma distribution with four categories (G4) substitution models for WNV and USUV, respectively (best-fit models identified by ModelFinder using the Bayesian information criterion). Time-scaled phylogenies were constructed from the consensus trees using the approximate maximum likelihood method in TreeTime [25] v0.11.3 with default settings including a stochastic method to resolve polytomies. The resulting trees were visualised using ggtree and associated packages, including treeio (v1.26.0) and tidytree (v0.4.6), assisted by the ape package (v5.8).

### Antibody testing

For detecting IgM and IgG antibodies, ELISA was employed, essentially as described previously [16]. In brief, microtitre plates were coated with formalin-inactivated WNV (strain NY99). Sera were tested in 10-fold serial dilution, and biotin-labelled anti-human IgM or IgG (Pierce Protein Biology Products, Thermo Fisher Scientific, Waltham, MA, United States) together with streptavidin−peroxidase (Sigma-Aldrich Inc., St. Louis, MO, United States) were used for detection.

Neutralisation assays were performed as described previously [16,18]. In brief, duplicates of serial twofold dilutions of heat-inactivated sera were mixed with 50–100 50% tissue culture infectious doses (TCID_50_) of WNV (strain NY99) or USUV (South African strain SAAR-1776), respectively, and incubated for 1 hour. Vero cells were added, and incubation was continued for 4–5 days. The presence of virus was assessed by the occurrence of a cytopathic effect. Neutralisation titres ≥ 20 were considered positive.

### Calculation of incidence rates

Annual incidence rates were determined for each affected Nomenclature of Units for Territorial Statistics (NUTS)-3 region by calculating the number of human infections per 100,000 residents. Population sizes were obtained from the public database of the National Statistical Institute of Austria [26]. Relative risk (RR) was computed as the ratio of the WNV incidence in 2024 to the average incidence recorded in years in which WNV cases were detected between 2009 and 2023 in the respective NUTS-3 region.

### Statistical analysis

Statistical analyses were performed with GraphPad Prism, version 9. Analyses of demographic and clinical characteristics were performed using Mann–Whitney U test and Fisher’s exact test with Bonferroni correction for multiple testing. The relationship between overall annual cases numbers of WNV and USUV was assessed with Spearman correlation.

## Results

### Incidence of human West Nile and Usutu virus infections in Austria

Since the first report of human WNV cases in Austria in 2009 [16], human infections have been detected in most years [1]. Up to 2024, a total of 97 WNV cases were diagnosed in this study. Of these, 74 were confirmed by PCR, eight by detection of WNV-specific IgM in CSF, and 15 by serological testing, further validated by neutralisation assays.

**Figure 1:**
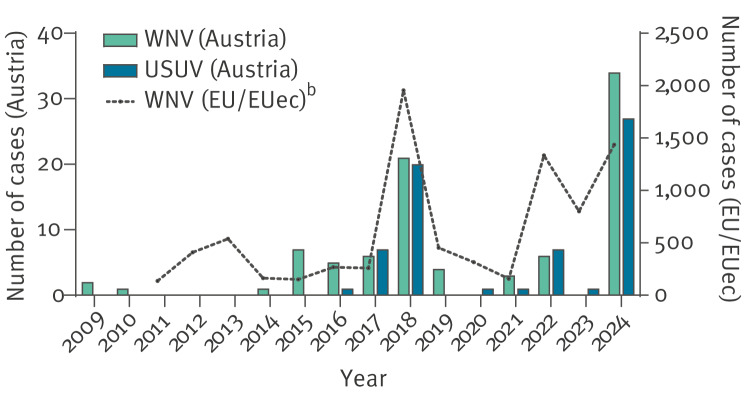
Annual numbers of autochthonous West Nile (n = 90)^a^ and Usutu virus cases (n = 65)^a^, Austria, 2009–2024

EU: European Union; EUec: EU enlargement countries; USUV: Usutu virus; WNV: West Nile virus.

^a^ WNV and USUV cases have been identified through nationwide patient diagnostics since 2009 and, since 2014, through screening of blood donors residing in or returning from Austrian provinces with endemic regions (Vienna, Lower Austria, Burgenland).

^b^ Cumulative data from EU countries (Austria, Bulgaria, Croatia, Cyprus, Czechia, France, Germany, Greece, Hungary, Italy, the Netherlands, Portugal, Romania, Slovenia, Slovakia, Spain) and EUec (Albania, Kosovo^§^, Montenegro, North Macedonia, Serbia, Türkiye) [1].

Seven of the 97 WNV cases reported had a travel history pointing to these as being imported. Thus, to assess the evolution of annual case numbers, a total of 90 WNV cases, which were most likely locally acquired in Austria were analysed by year of detection. This analysis revealed considerable variations, with annual WNV case numbers in Austria following approximately the same temporal tendencies as aggregate numbers of cases recorded every year in the EU and EU-enlargement countries [1] (Figure 1). While only few annual WNV cases (˂ 10) were recorded between 2009 and 2017, a marked increase in their numbers occurred in 2018 [18]. Thereafter, annual values remained within prior levels until 2024, when another strong surge in case numbers took place (Figure 1).

**Figure 2:**
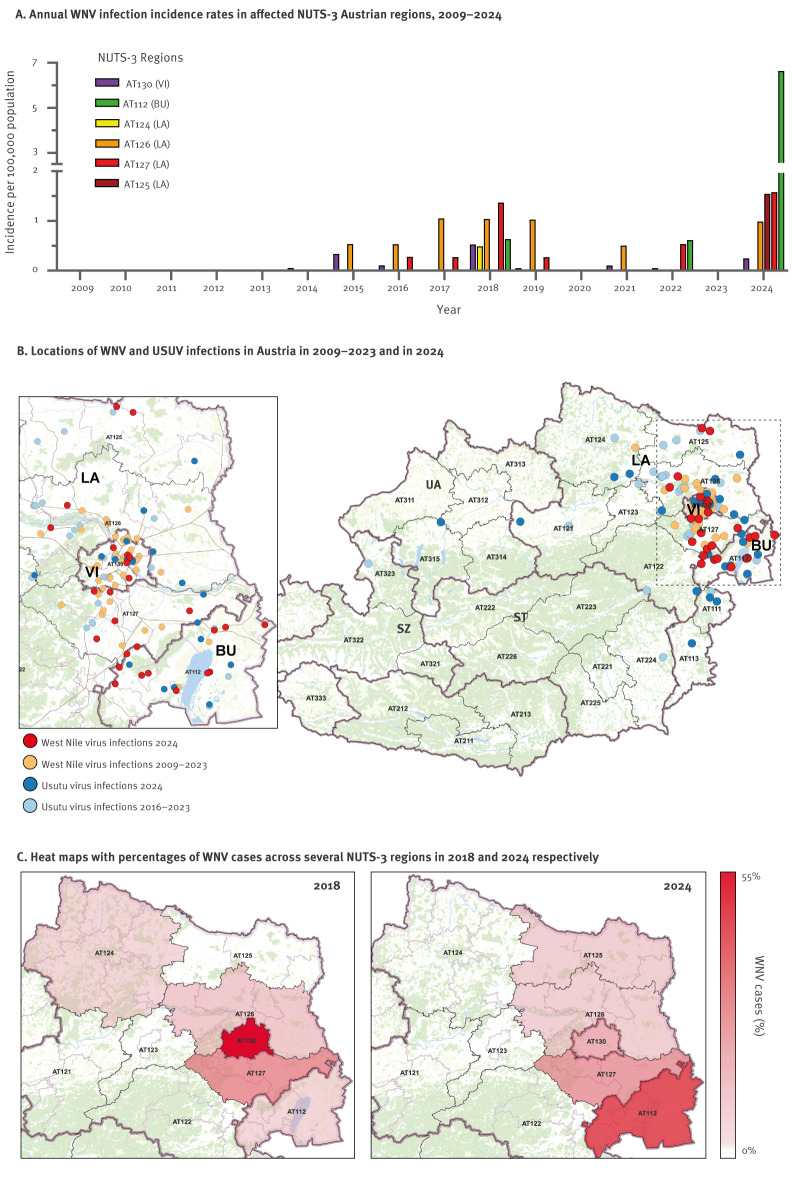
(A) Annual incidence rates of PCR-confirmed WNV cases by most likely place of exposure, (B) geographic distribution of autochthonous WNV and USUV cases and (C) heat maps with percentages of WNV cases across several WNV-affected NUTS-3 regions in 2018 and 2024 respectively, Austria, 2009–2024 (n = 71 WNV cases; n = 65 USUV cases)

AT112: northern Burgenland; AT124: Waldviertel; AT125: Weinviertel; AT126: northern part of Vienna-surrounding area; AT127: southern part of Vienna-surrounding area; AT130: Vienna; BU: Burgenland federal province; LA: Lower Austria federal province; ST: Styria federal province; SZ: Salzburg federal province; UA: Upper Austria federal province; USUV: Usutu virus; VI: Vienna federal province; WNV: West Nile virus.

In 2024 alone, 37 WNV cases in total were notified in Austria, in accordance with the EU case definition. Among these, WNV was confirmed by PCR in 29, four additional cases were confirmed by WNV-specific IgM in CSF, and a further four by high-titre IgM and IgG with confirmatory virus-specific neutralisation tests (Table 1). Among the 37 laboratory-confirmed cases, 34 had reported no travel history outside Austria in the 14 days prior to symptom onset or blood donation and were thus considered locally acquired. During 2024, the north region of the Burgenland province, i.e. northern Burgenland, was particularly affected by WNV, accounting for 16 cases (Figure 2).

**Table 1:** Detailed information of human West Nile virus and Usutu virus infections, Austria, 2024 (n = 64 cases)

Characteristics		Clinical cases	Blood donors	Total
**WNV**				
Total		26	11	37
Location	Autochthonous	23	11	34
	Imported	3	0	3
Laboratory confirmation	PCR	18	11	29
	Serology	8	0	8
Clinical characteristics	Asymptomatic	0	5	5
	WNF	7	6	13
	WNND	19	0	19
	Hospitalised	23	0	23
**USUV**				
Total		0	27	27

USUV: Usutu virus; WNF: West Nile fever; WNND: West Nile neuroinvasive disease; WNV: West Nile virus.

In addition to USUV screening in blood donors, testing for USUV was attempted for all reported WNV cases to minimise potential issues due to cross-reactivity in WNV-antibody testing. For three cases, USUV testing could not be performed due to insufficient sample volume. For the remainder WNV cases, USUV test results were negative, except for one case in 2018 that was confirmed to be co-infected with both WNV and USUV.

In total, 65 USUV infections were detected throughout the study period, including 57 confirmed by PCR and eight by neutralisation assays. Figure 1 shows a marked increase in USUV cases in 2018, followed by low annual case numbers (˂ 10) during 2019–2023 and a raise in 2024. In 2024, 27 local USUV cases were identified among blood donors (with 24 confirmed by PCR and 3 by neutralisation assays).

To examine whether regional changes occurred in endemic areas, we estimated the incidence rates for all affected NUTS-3 regions within the Austrian federal provinces of Vienna, Lower Austria, and Burgenland. This was determined based on PCR-confirmed WNV cases to ensure high specificity and temporal accuracy, allowing for precise spatiotemporal mapping of transmission dynamics (Figure 2A). As three PCR-confirmed WNV cases were imported, a total of 71 PCR-confirmed WNV autochthonous were included in this analysis.

The region of northern Burgenland (AT112) recorded the most pronounced increase in WNV incidence rate in 2024. Incidence rates in this region were low until 2023 (≤ 0.63 per 100,000) and then rose to 6.64 per 100,000 in 2024, surpassing the rates in the Vienna (AT130; 0.25 per 100,000) and Lower Austrian regions (AT124–127; 0–1.58 per 100,000; Figure 2A,B). While a moderate increase in WNV incidence was observed in the southern part of Vienna-surrounding area (AT127), the RR for 2024 compared with previous years was markedly higher in northern Burgenland (RR = 3.46 for AT127 vs RR = 10.65 for AT112). The geographical shift in WNV activity in Austria was especially evident when comparing 2018 and 2024, the 2 years with the highest case counts (Figure 2C). 

Although USUV showed a wider distribution and was occasionally detected in the federal provinces of Styria, Upper Austria, and Salzburg, most cases occurred in the same transmission hotspots as WNV (Figure 2B). In particular, northern Burgenland also experienced a marked increase in USUV incidence, reaching 4.2 cases per 100,000 in 2024, compared with 0.64–1.27 per 100,000 in previous years in which infections were detected. In Austria overall, annual USUV case numbers in blood donors correlated with WNV cases (Spearman r = 0.73, p = 0.033). Analysis of all WNV cases meeting the EU case definition, including serologically diagnosed cases, revealed a similar regional distribution, confirming the geographical shift towards northern Burgenland, identified with PCR-confirmed cases, as shown in Supplementary Figure 1.

### Sequence information

Sequence-based lineage information using short WNV C/prM gene fragments was obtained for a total of 66 WNV cases between 2009 and 2024. Of these, all locally acquired WNV cases (n = 63), including 25 sequenced in 2024, were identified as WNV lineage 2. Of the three imported WNV cases detected in 2024, two were lineage 2, and one was lineage 1. The lineage 1 strain, detected in a traveller returning from India, showed 98.9% sequence similarity to a human isolate from Kerala, India (GenBank accession number: KC601756).

**Figure 3:**
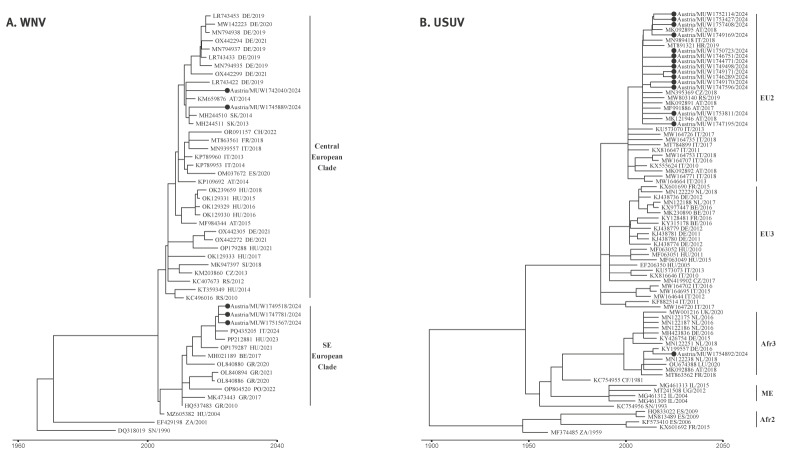
Time-resolved phylogenetic analysis of West Nile virus and Usutu virus sequences from Austria, 2024

Afr2: Africa-2; Afr3: Africa-3; EU2: Europe-2; EU3: Europe-3; ME: Middle East; SE: southeastern European clade.

We performed a time-resolved phylogenetic analysis of 1,880 nt sequences obtained from five Austrian patients in 2024 compared with reference sequences from across Europe, including those previously reported from Austria. We found that all five sequences clustered within subclades of the monophyletic clade of WNV lineage 2 (clade 2d-1) (Figure 3A) that has been circulating in Europe since its first detection in Hungary in 2004 [23]. Two of the sequences were in the central European subclade, whereas three were in the southeastern European subclade of WNV lineage 2, following the nomenclature by Chaintoutis and Mencatelli [12,27], marking the first record of the southeastern European subclade of WNV-2 in Austria.

Sequences of (A) WNV and (B) USUV obtained from individuals in Austria in 2024 (black circles) are each compared with WNV and USUV reference sequences and sequences previously described from Austria. The respective relationships among (A) WNV sequences and (B) USUV sequences are assessed by maximum likelihood phylogenetic analysis, with branch lengths of the phylogenetic trees adjusted according to the viruses’ sampling dates (shown in years on the X-axis). The inferred molecular clock rates are 0.000373 substitutions per site per year for WNV (r^2^ = 0.76) and 0.000213 substitutions per site per year for USUV (r^2^ = 0.14) based on a root-to-tip regression.

**A.** The WNV time-resolved phylogenetic tree includes five 1,880 nt sequences obtained from patients in Austria in 2024. All five sequences belong to the WNV-2 clade, with two clustering within the central European subclade and three within the southeastern (SE) European subclade, following the nomenclature and analysis of Mencatelli et al. 2023 [12] and Chaintoutis et al. 2019 [27].

**B.** The USUV phylogenetic tree includes major USUV genotypes indicated next to monophyletic clades (EU2, EU3, Afr2, Afr3, ME). A non-redundant set of 15 new sequences obtained from blood donors in Austria in 2024 are analysed and 14 of these cluster within the EU2 clade and one within the Afr3 clade.

Phylogenetic analysis of USUV detected in 2024 was performed with 15 non-redundant sequences of the 24 amplified by RT-PCR, as well as sequences from across Europe, including those previously reported from Austria. All sequences from Austria in 2024 grouped within the ‘Europe-2’ clade, except for one sample in the ‘Africa-3’ clade which had 100% sequence identity to a strain identified in an Austrian blood donor in 2018 [18] (Figure 3B).

### Demographic and clinical characteristics of West Nile and Usutu virus cases

Of the 37 WNV cases (19 male/18 female; median age: 62 years; range: 18–88) in 2024, 26 were detected through clinical evaluation and 11 by blood donor screening (Table 1). Nineteen cases (10 male/9 female; median age: 66 years; range: 32–88) had WNND with three of them imported and 16 autochthonous. A total of 13 (6 male/7 female; median age: 57 years; range: 18–65) had WNF, and five were asymptomatic (3 male/2 female; median age: 49; range: 45–63). Among clinical cases, 18 were diagnosed by PCR and eight by serology; all blood donor cases were PCR-confirmed (Table 1).

Neurological symptoms in PCR-confirmed WNND cases (n = 14 with 11 autochthonous) included confusion, headache, vertigo, decrease of vigilance, ataxia, seizure and coma (Table 2). Among PCR-confirmed WNF cases (four reported by clinicians, six identified through blood donation screening), the most common symptoms were fever, headache, exanthema, fatigue, myalgia and/or arthralgia. Based on information from 2019 to 2024, when systematically-documented clinical data were available, we observed that in patients with WNF, myalgia/arthralgia was more frequent than in those with WNND (5 of 16 vs 0 of 16; Fisher’s exact test; p = 0.043), and exanthema also tended to occur more often in WNF (9 of 16 vs 3 of 16; p = 0.066) among PCR-confirmed cases, consistent with previous studies [28-31].

**Table 2:** Characteristics of West Nile virus infections detected through clinical evaluation, Austria, 2024 (n = 26 cases)

Syndrome Assay	Case count	Age in years	Sex	Hospitalisation	Calendar week of symptom onset	Main symptoms	WNV				USUV	
							PCR^a^	Blood		CSF	Blood	
								IgM	NT	IgM	NT	PCR^a^
WNND												
PCR^b^	1	81	M	Yes	30	Fever, rash, disorientation, somnolence	Pos	Pos	120	Pos	Neg	Neg
	2	44	M	Yes	31	Fever, headache, rash, ataxia, vertigo	Pos	Pos	960	Pos	40	Neg
	3^c^	74	M	Yes	31	Fever, ataxia, headache	Pos	Pos	120	Pos	160	Neg
	4	77	F	Yes	32	Fever, headache, delirium, speech impairment	Pos	Pos	320	Pos	160	Neg
	5	65	M	Yes	32	Fever, tremor	Pos	Pos	320	Pos	20	Neg
	6	66	M	Yes	34	Diarrhoea, weakness, coma	Pos	Pos	320	Pos	160	Neg
	7	63	F	Yes	34	Fever, confusion^d^	Pos	Pos	120	n.a.	Neg	Neg
	8	88	F	Yes	33	Fever, confusion	Pos	Pos	480	Pos	160	Neg
	9	32	M	Yes	34	Headache, exanthema, meningitis	Pos	Pos	640	Pos	20	Neg
	10	69	F	Yes	35	Confusion, memory impairment	Pos	Pos	240	Pos	Neg	Neg
	11	77	F	Yes	36	Vigilance impairment, coma	Pos	Pos	320	Pos	60	Neg
	12	51	M	Yes	37	Fever, confusion, seizure, vigilance impairment^e^	Pos	Pos	960	BL	Neg	Neg
	13	82	M	Yes	35	Fever, mild confusion^f^	Pos	Pos	160	Pos	20	Neg
	14	64	F	Yes	43	Fever, headache, vertigo, seizure^g^	Pos	Neg	20	Neg	Neg	Neg
Serology^h^	15	76	M	Yes	33	Fever, confusion, ataxia	Neg	Pos	160	Pos	60	Neg
	16	41	F	Yes	35	Fever, headache, exanthema, dizziness, cognitive impairment	Neg	Pos	120	Neg	30	Neg
	17	47	F	Yes	34	Malaise, nausea, paresis	Neg	Pos	160	Pos	20	Neg
	18	66	M	Yes	33	Headache, neck stiffness, change of behaviour	Neg	Pos	320	Pos	80	Neg
	19	55	F	Yes	35	Headache, fever, confusion	Neg	Pos	160	Pos	40	Neg
WNF												
PCR^b^	1	36	F	No	35	Fever, headache, exanthema, fatigue	Pos	Pos	480	n.a.	40	Neg
	2	59	F	Yes	35	Fever, headache, myalgia/arthralgia	Pos	Pos	240	BL	240	Neg
	3	65	M	Yes	35	Fever, headache, fatigue, exanthema, weakness	Pos	BL	Neg	Neg	Neg	Neg
	4	64	M	Yes	37	Confusion, change of behaviour^g,i^	Pos	Pos	20	Neg	Neg	Neg
Serology^h^	5	62	F	No	33	Fatigue, myalgia/arthralgia, exanthema	Neg	Pos	320	n.a.	120	Neg
	6	40	F	No	35	Fever, exanthema, fatigue, nausea	Neg	Pos	1280	n.a.	160	Neg
	7	62	F	Yes	35	Fever, exanthema, diarrhoea^g^	Neg	Pos	480	n.a.	120	Neg

BL: borderline; CSF: cerebrospinal fluid; F: female; Hosp.: hospitalisation; M: male; n.a.: not assessed; Neg: negative; NT: neutralisation test; Pos: positive; USUV: Usutu virus; WNF: West Nile fever; WNND: West Nile neuroinvasive disease; WNV: West Nile virus.

^a^ PCR performed in serum, whole blood, urine, or CSF.

^b^ Confirmed by PCR detection of WNV RNA.

^c^ In this PCR-confirmed WNV case, stronger USUV than WNV neutralisation was observed despite a negative USUV PCR result.

^d^ Pancreatic carcinoma comorbidity.

^e^ Hodgkin lymphoma comorbidity.

^f^ Neuroborreliosis comorbidity.

^g^ Immunosuppression comorbidity.

^h^ Serological diagnosis according to European Union case definitions [19].

^i^ Multiple sclerosis comorbidity.

Overall, 23 of 37 WNV cases in 2024 required hospitalisation (all WNND patients and 3 WNF patients), with no fatalities reported (Table 2). By contrast, three of 27 USUV infections (18 male/9 female; median age: 59 years; range: 20–69) identified by blood donor screening showed symptoms (arthralgia, fatigue, respiratory symptoms, influenza-like illness, and pruritus) (Table 3).

**Table 3:** Characteristics of West Nile virus (n = 11 cases) and Usutu virus infections (n = 27 cases) detected by blood donation screening, Austria, 2024

Case count	Age in years	Sex	Calendar week of blood donation	Main symptoms	WNV			USUV	
					PCR	IgM	NT	PCR	NT
WNV									
1	45	M	29	Asymptomatic	Pos	Neg	n.a.	Neg	n.a.
2	61	M	29	Asymptomatic	Pos	Neg	n.a.	Neg	n.a.
3	63	M	30	Asymptomatic	Pos	Neg	n.a.	Neg	n.a.
4	55	M	31	Headache	Pos	Neg	n.a.	Neg	n.a.
5	54	M	31	Nausea, fatigue	Pos	Pos	n.a.	Neg	n.a.
6	49	F	31	Asymptomatic	Pos	Neg	n.a.	Neg	n.a.
7	18	F	34	Fever, headache, exanthema	Pos	Neg	n.a.	Neg	n.a.
8	55	M	34	Exanthema, fatigue	Pos	Neg	n.a.	Neg	n.a.
9	57	M	35	Fever, fatigue, headache, myalgia/arthralgia	Pos	Neg	n.a.	Neg	n.a.
10	27	F	35	Fever, headache, exanthema, myalgia/arthralgia	Pos	Neg	n.a.	Neg	n.a.
11	45	M	35	Asymptomatic	Pos	Neg	n.a.	Neg	n.a.
USUV									
1	54	M	29	Asymptomatic	Neg	Neg	n.a.	Pos	n.a.
2	65	M	30	Asymptomatic	Neg	Neg	n.a.	Pos	n.a.
3	53	F	30	Myalgia/arthralgia, fatigue, cough	Neg	Neg	n.a.	Pos	n.a.
4	48	F	31	Asymptomatic	Neg	Neg	Neg	Neg	40
5	60	M	31	Asymptomatic	Neg	Neg	n.a.	Pos	n.a.
6	68	F	32	Asymptomatic	Neg	Neg	n.a.	Pos	n.a.
7	52	F	32	Asymptomatic	Neg	Neg	n.a.	Pos	n.a.
8	60	M	32	Asymptomatic	Neg	Neg	n.a.	Pos	n.a.
9	63	M	32	Asymptomatic	Neg	Neg	n.a.	Pos	n.a.
10	49	F	33	Asymptomatic	Neg	Neg	n.a.	Pos	n.a.
11	59	M	33	Asymptomatic	Neg	Neg	n.a.	Pos	n.a.
12	46	M	33	Asymptomatic	Neg	Neg	n.a.	Pos	n.a.
13	53	M	34	Asymptomatic	Neg	Neg	n.a.	Pos	n.a.
14	62	M	34	Asymptomatic	Neg	Neg	n.a.	Pos	n.a.
15	66	M	34	Pruritus	Neg	Neg	n.a.	Pos	n.a.
16	20	F	35	Asymptomatic	Neg	Neg	n.a.	Pos	n.a.
17	60	M	36	Asymptomatic	Neg	BL	30	Neg	120
18	55	M	36	Influenza-like illness, malaise	Neg	Neg	n.a.	Pos	n.a.
19	52	M	36	Asymptomatic	Neg	Neg	n.a.	Pos	n.a.
20	69	M	36	Asymptomatic	Neg	Neg	n.a.	Pos	n.a.
21	61	F	36	Asymptomatic	Neg	Neg	n.a.	Pos	n.a.
22	59	F	37	Asymptomatic	Neg	Neg	n.a.	Pos	n.a.
23	59	M	37	Asymptomatic	Neg	Neg	n.a.	Pos	n.a.
24^a^	60	F	37	Asymptomatic	Neg	Pos	n.a.	Pos	n.a.
25	67	M	38	Asymptomatic	Neg	Neg	n.a.	Pos	n.a.
26	46	M	38	Asymptomatic	Neg	Neg	n.a.	Pos	n.a.
27	54	M	42	Asymptomatic	Neg	Neg	Neg	Neg	60

BL: borderline; F: female; M: male; n.a.: not assessed; Neg: negative; NT: neutralisation test; Pos: positive; USUV: Usutu virus; WNV: West Nile virus.

^a^ This USUV case also exceeded the WNV IgM threshold.

Among all 37 WNV cases from 2024, WNV RNA was most frequently detected in whole blood samples (15 of 22), followed by urine (10 of 23), serum (8 of 24) and CSF (2 of 15). Notably, follow-up testing of whole blood and/or urine detected WNV RNA in eight of 14 patients whose serum initially tested negative by RT-PCR. Among 38 NAT-positive blood donors, 11 were confirmed as WNV by RT-PCR, while 27 were confirmed as USUV (24 by RT-PCR, and 3 via neutralisation assay in follow-up samples) (Table 3).

## Discussion

We investigated the epidemiological patterns of WNV and USUV infections in Austria over a 15-year period. Multiple factors have been documented to influence the occurrence of WNV and USUV infections. These include climatic changes as well as ecological factors like agricultural density that can influence avian and mosquito populations [11,32].

In 2024, a notable rise in WNV incidence was observed in Austria, particularly in northern Burgenland — an area in eastern Austria with historically low incidence of WNV. In this region, cases were concentrated in an agricultural sector near Lake Neusiedl, Austria's largest lake. This wetland area located very close to the Hungarian and close to Slovak borders provides a vital habitat for both migratory and resident bird species, like other European wetlands with endemic WNV [28,29]. Moreover, the climatic conditions in 2024, characterised by a warm winter and above-average summer temperatures, and below-average precipitation levels [33], likely increased vector abundance and virus transmission [34]. Such regional expansions align with patterns observed in other parts of Europe, suggesting a broader trend of expanding affected areas [28,35].

Lineage determination confirmed that autochthonous WNV strains were all lineage 2. Phylogenetic comparisons showed that the 2024 sequences were similar to those identified from humans, birds, and mosquitoes from previous years in Europe, in agreement with earlier reports [27,36]. Notably, our time-resolved phylogenetic analyses provide the first evidence of the southeastern European clade of WNV-2 in Austria (Figure 3), whereas both Europe-2 and Africa-3 clades of USUV have been detected previously in Austrian patients and birds [18,37]. The limitations of our study include the reliance on relatively short sequences to confirm general similarities to reference strains and low viral loads that limited phylogenetic resolution; obtaining longer virus sequences would provide more robust phylogenetic analysis. Moreover, WNV sequences from bird or mosquito surveillance with detailed geographic data and high viral loads would strengthen phylogeographic analyses to determine whether introduction of the southeastern European clade of WNV-2 was associated with the observed increase in incidence in Burgenland.

The 2024 increase in WNV was concomitant with a substantial rise in human USUV cases, with 27 cases detected in blood donors, representing the highest recorded case count in Austria to date. Furthermore, throughout the observed period, we observed a strong correlation between human WNV and USUV cases in blood donors, which was expected and has been attributed to similar factors that drive their enzootic maintenance and transmission [4,34]. Compared with the high rate of symptomatic disease in WNV infected blood donors (6/11) and the high number of patients with WNND (n = 19), only three of 27 donors infected with USUV reported mild symptoms, in line with the overall lower pathogenicity of USUV [38]. However, USUV infection can occasionally result in severe neurological disease [5,39,40], underscoring the need for further investigation into its impact on human health.

Surveillance of WNV that includes differential testing for USUV can provide important insights into USUV epidemiology and clinical manifestations. In our study, most WNV and USUV cases were identified by virus-specific PCR, supported by virus sequencing. However, in some instances, particularly in clinical WNV cases, diagnosis relied on serology, which requires IgM detection in CSF or IgM/IgG in serum confirmed by virus-specific neutralisation assays, in accordance with the EU case definition [19]. While WNV IgM testing showed high sensitivity in our cohort, one acute USUV case also exceeded the WNV IgM threshold. Similar findings, including evidence of cross-reactivity in neutralising antibodies have been reported by others [41], underscoring the importance of conducting parallel virus neutralisation assays for both WNV and USUV [20]. In one PCR-confirmed WNV case, stronger USUV than WNV neutralisation was observed despite a negative USUV PCR result, suggesting possible pre-existing immunity to USUV. Considering the increasing co-circulation of WNV and USUV, improved methods for refining serological differentiation [42] will be important to support surveillance under evolving epidemiological conditions.

## Conclusion

Our analysis shows a rise in WNV cases in a previously only sporadically affected area, including an increase in WNND and infected blood donors. This study illustrates the growing public health impact of WNV in Austria and underlines the importance of continued surveillance for monitoring the changing patterns of WNV transmission, ensuring timely public health responses to future WNV outbreaks. Integrating USUV testing into WNV surveillance can mitigate the likelihood of donor-derived USUV transmission in areas where both viruses co-circulate and enhance our understanding of the epidemiology and clinical relevance of USUV.

## Data Availability

West Nile virus and Usutu virus sequences have been uploaded to GenBank with accession numbers PQ785861-PQ785893.

## ^§^Note

This designation is without prejudice to positions on status and is in line with United Nations Security Council Resolution 1244/99 and the International Court of Justice Opinion on the Kosovo Declaration of Independence.

## Supplementary Data

430000 Supplementary Material
