# Supplementary material for: Increased incidence of human West Nile and Usutu infections in Austria, 2024: analysis of data from 2009 to 2024
Source: Euro Surveill. 2026 Jan 8;31(1):2500260. doi: 10.2807/1560-7917.ES.2026.31.1.2500260 (PMC12862289; doi:10.2807/1560-7917.ES.2026.31.1.2500260)
Supplement: Supplementary Material [file Supplementary_Material_2500260.pdf]

Supplementary Material

Increased incidence of human West Nile and Usutu infections in Austria, 2024: analysis of data from 2009 to 2024

This supplementary material is hosted by *Eurosurveillance* as supporting information alongside the article "Increased incidence of human West Nile and Usutu infections in Austria, 2024: analysis of data from 2009 to 2024", on behalf of the authors, who remain responsible for the accuracy and appropriateness of the content. The same standards for ethics, copyright, attributions and permissions as for the article apply. Supplements are not edited by *Eurosurveillance* and the journal is not responsible for the maintenance of any links or email addresses provided therein.

Table S1: Details of PCR-confirmed symptomatic human West Nile virus infections, Austria, 2019-2024 (n = 32 cases)

Patient characteristics (2019-2024)			
	**WNND (n=16)**	**WNF (n=16)**	
Age	68 (32-88)	54 (18-68)	p<0.001
Sex (F/M)	7/9	7/9	
Blood donor	0	9	
Hospitalized	16	2	
**Symptoms other than neurologic symptoms**			
Fever	11	7	
Exanthema	3	9	p=0.066
Headache	6	8	
Malaise	9	7	
Gastrointestinal symptoms	2	1	
Myalgia/Arthralgia	0	5	p=0.043

WNND: West Nile neurologic disease; WNF: West Nile fever.

Figure S1: Geographic distribution of autochthonous West Nile cases and incidence rates by most likely place of exposure in Austria, 2009-2024 (n = 90 cases)

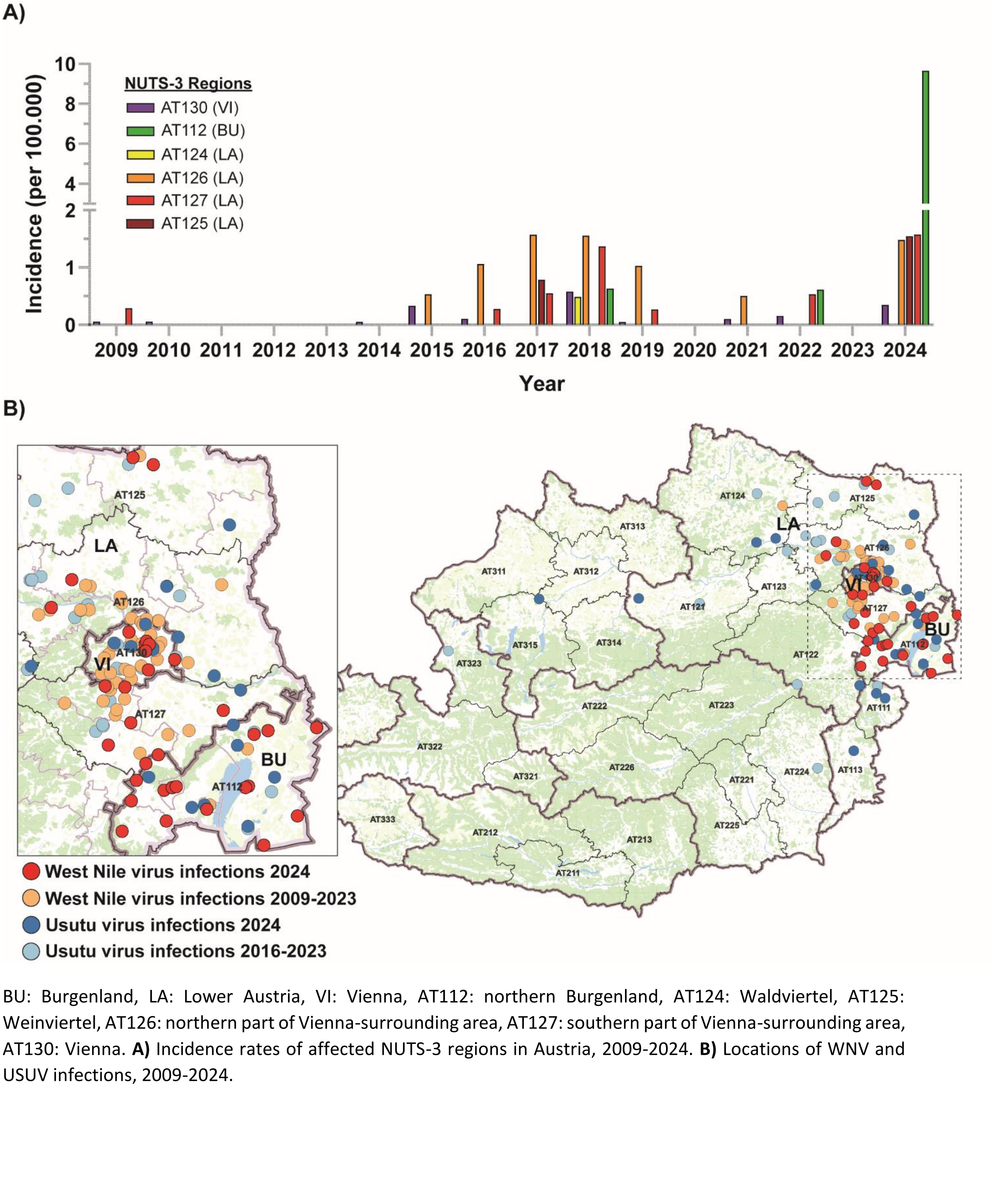

BU: Burgenland, LA: Lower Austria, VI: Vienna, AT112: northern Burgenland, AT124: Waldviertel, AT125: Weinviertel, AT126: northern part of Vienna-surrounding area, AT127: southern part of Vienna-surrounding area, AT130: Vienna. **A)** Incidence rates of affected NUTS-3 regions in Austria, 2009-2024. **B)** Locations of WNV and USUV infections, 2009-2024.
